# Diterpenes and triterpenes show potential as biocides against pathogenic fungi and oomycetes: a screening study

**DOI:** 10.1007/s10529-023-03438-z

**Published:** 2023-11-01

**Authors:** Sylwia Adamczyk, Satu Latvala, Anna Poimala, Bartosz Adamczyk, Tuija Hytönen, Taina Pennanen

**Affiliations:** Natural Resources Institute Finland (Luke), Latokartanonkaari 9, 00790 Helsinki, Finland

**Keywords:** Colophony, Fungicide, Reactive oxygen species, Resin

## Abstract

The aim was to screen di- and triterpenes as potential biocides against fungal pathogens (*Alternaria* sp., *Fusarium avenaceum*, *F. sambucinum*, *Botrytis cinerea*, *Botryotina fuckeliana*, *Mycocentrospora acerina*, *Cylindrocarpon* sp.) and *oomycetes (Phytophthora cactorum*, *P. fragariae)*. *Results* We measured the antifungal activity of terpenes by estimating the growth area, ergosterol content and level of lipid peroxidation. Fungi and oomycetes were grown on solid media in Petri dishes. As a positive control, we used a common synthetic fungicide, fosetyl-Al. Di- and triterpenes showed promising potential as biocides against most of the studied species. The responses of fungi and oomycetes were dependent on the specific type of terpenes and identity of the fungi. Compared to synthetic fungicide, terpenes were equally effective as antifungal agents and even more effective for some species, especially for oomycetes. The terpene mode of action includes inhibition of ergosterol synthesis and increased lipid peroxidation. *Conclusions* Di- and triterpenes, natural compounds that are very abundant in northern countries, are excellent candidates for biocides.

**Supplementary Information:**

The online version contains supplementary material available at 10.1007/s10529-023-03438-z.

## Introduction

According to the Food and Agriculture Organization of the United Nations, pathogens are responsible for destroying more than one-fifth of crops produced worldwide each year. In Northern Europe, the main cereal crops, oat (*Avena sativa*) and barley (*Hordeum vulgare L*.), suffer from severe *Fusarium sp*. infections (Hietaniemi et al. 2016). *Botrytis cinerea* (teleomorph: *Botryotinia fuckeliana*), another common plant pathogen, attacks over 200 crop hosts worldwide (Williamson et al. 2007). Other fungal species, such as *Mycocentrospora acerina*, *Alternaria* spp., and *Cylindrocarpon* spp., are pathogens causing pre- and postharvest damage to agricultural products, and they can produce mycotoxins harmful to humans (Iwen et al. 2000; Patriarca et al. 2014; Cheung et al. 2020). In addition to fungi, oomycetes affect plant yield, i.e., *Phytophthora* spp., common pathogens of potato (*Solanum tuberosum)* and strawberry (*Fragaria Ananassa*) (Vleeshouwers et al. 2011; Toljamo et al. 2016; Adams et al. 2020). To address concerns related to food production and food safety, synthetic pesticides have been developed. However, pesticides exhibit adverse effects on nontarget organisms and human health (Pathak et al. 2022). Moreover, pathogens may develop resistance against pesticides (Tang and Maggi 2021). Thus, there is an urgent need to replace pesticides with less harmful substances. These biocides should originate from related ecosystems and should target the most common pathogens to avoid side effects (Isman 2006). Although studies have affirmed the antifungal properties of numerous natural compounds, little progress has been achieved in developing a human-safe biocide with a wide spectrum against common pathogens.

Plants have evolved multiple mechanisms against pathogens, including plant secondary metabolites (PSMs, e.g., Harborne, 1997). PSMs are especially abundant in long-lived boreal forest trees, including Norway spruce (*Picea abies*) and Scots pine (*Pinus sylvestris*) (Horwath 2015). Terpenes are the most versatile group of PSMs and consist of volatile (monoterpenes, sesquiterpenes) and nonvolatile compounds (higher terpenes: di-, tri-, and polyterpenes). Laboratory studies suggest that volatile and nonvolatile conifer terpenes can affect fungal growth and germination (Adamczyk et al. 2013; Kusumoto et al. 2014). Less is known about the effect of different classes of terpenes on pathogens, and the mechanisms of action employed by individual terpenes against pathogens are not well understood. Terpenes may deteriorate fungal activity through reactions with enzymes, e.g., thymol and limonene (volatile terpenes) decrease the activity of methyl esterase and cellulase (Marei et al. 2012). Monoterpenes and higher terpenes decrease the activity of numerous enzymes, including acid phosphatase, chitinase and protease (Adamczyk et al. 2015). Another mechanism of action was proposed for phenolics and aldehydic terpenes; these compounds were shown to decrease the biosynthesis of ergosterol, an ingredient of fungal cell walls, and increase the peroxidation of lipids (Kumari et al. 2019). High levels of lipid peroxidation promote loss of integrity in the plasma membrane, eventually leading to cell death (Avis et al. 2007).

The aim of this study was to screen di- and triterpenes that are present in coniferous wood in significant quantities as potential biocides against a wide group of common fungal and oomycete pathogen species. Fungi were selected to represent a wide variety of taxonomic backgrounds (Ascomycetes, Oomycetes) and could cause diseases in a large selection of host plants (Table 1). As a positive control, we used a synthetic fungicide. To estimate the effect of treatments on fungi, we measured fungal growth area, ergosterol concentration and lipid peroxidation.

**Table 1:** Fungal and oomycete cultures used in the experiments

ID	Fungus	Phylum	Host	Disease	References
FAV/1, FAV/2	*Fusarium avenaceum*	Ascomycota	Carrot	Fusarium (dry) rot	Collected by Natural Resources Institute Finland (Luke) 2018
FSAM/1	*Fusarium sambucinum*	Ascomycota	Carrot	Fusarium (dry) rot	Collected by Luke 2018
BC/1	*Botrytis cinerea*	Ascomycota	Carrot	Grey mould	
CYL/1, CYL/2	*Cylindrocarpon* sp.	Ascomycota	Carrot	Pit symptoms on carrot root	
MAC/1, MAC/2	*Mycocentrospora acerina*	Ascomycota	Carrot	Liquorice rot	
Alternaria sp.1	*Alternaria* sp*.*	Ascomycota	Carrot	Black rot	
B.f.VT7-19 B.f.VS34-5	*Botryotinia fuckeliana*	Ascomycota	Picea abies seedling root	Grey mould	Collected by Luke 2013
PhF06/19, PhF22/19	*Phytophthora cactorum*	Oomycota	Fragaria x ananassa 'Polka'	Crown rot	(Poimala et al. 2021)
BBAL1, BBAL1/2	*Phytophthora fragariae*	Oomycota	Fragaria x ananassa	Red stele	Collected by Julius Kühn Institut, Germany 1983

## Materials and methods

### Fungal pathogens

Fungal pathogens (Table 1) were obtained from the culture collection of Natural Resources Institute Finland.

### Terpenes and fungicide

Terpenes (beta-sitosterol, abietic acid, colophony) were purchased from Sigma‒Aldrich. Beta-sitosterol represents triterpenes, and abietic acid is a diterpene. We collected resin from spruce trees. Mature spruce trees, several individuals, were artificially wounded, and containers were placed below these wounds to collect resin (as in Uusitalo et al. 2008). Resin contains a mixture of a wide set of terpenes and fats, fatty acids, steryl esters, sterols and waxes (Bäck et al. 2010). Colophony is a mixture of diterpenes, and the used colophony was characterized as in Adamczyk et al. (2011) and contains abietic acid (37.7%), palustric acid (22.2%), neoabietic acid (18.4%), pimaric acid (8.4%), dehydroabietic acid (7.6%) and isopimaric acid (5.7%). All terpenes were mixed with ethanol (99%) at a 1% concentration.

As a fungicide, we used Aliette 80WG (Berner Oy). It contains the active compound fosetyl-Al ([C_2_H_5_OP(H)O_2_]_3_Al), which is an organophosphorus compound often used as a fungicide. This fungicide was chosen because it acts against fungi and oomycetes and only a few resistance cases have been reported (FRAC Code List © 2022). The fungicide was used according to the recommendations of the manufacturer at a concentration of 0.3%.

### Cultivation of fungi and treatments

The efficacy of terpenes against pathogens was tested with the poisoned food technique. Fungi and oomycetes were cultivated in Petri dishes (diameter 9 cm) as solid cultures on PDA medium (Sigma‒Aldrich) except *Phytophtora fragariae,* which was cultivated on solid modified orange serum (MOS) medium (Müller et al. 1994). Each fungus and oomycetes was cultivated with 3 replicates.

One millilitre of each treatment (control—99% ethanol; 1% terpenes in 99% ethanol; fungicide) was added and distributed on the plate with nutrient medium and agar, and after 2 h, the ethanol had evaporated from the medium (under a laminar cabinet). Then, fungi were inoculated from agar plugs (one or two plugs of agar into the middle of the plate) and grown at 20 °C in darkness for 9 days (all of the species). Next, we included additional controls with only fungi on the plates without any terpene or solvent (ethanol) and incubated at the same conditions (9 days, 20 °C in the darkness) to ensure that the fungi used for the study were viable and growing normally with no negative effect due to ethanol. The experiment was repeated 5 times with each fungus/oomycete.

The minimum inhibitory concentration and minimum fungicidal concentration were estimated under similar conditions as described above with 100, 200, 500, 1000, 2000, 5000, and 10,000 ppm of terpenes.

### Fungal and oomycete growth and biomass (ergosterol concentration)

To estimate the growth area of fungi (in cm^2^), we used ImageJ software (Schneider et al. 2012), and images of the fungi and oomycetes grown in Petri dishes are shown in the Supporting Information.

Changes in fungal biomass were estimated by ergosterol measured with the classic high-performance liquid chromatography (HPLC) method (Frostegård and Bååth 1996) as described by Adamczyk et al. (2019). Briefly, 25 mg of material (freeze-dried content of Petri dish) was extracted with 10% KOH in methanol. After vortexing and sonication for 15 min, the samples were incubated for 1 h at 70 °C. After cooling, 0.5 ml of H_2_O and 1 ml of cyclohexane were added, and the samples were then vortexed and centrifuged. The upper phase (cyclohexane) was transferred to the next tube, and the extraction was repeated. The combined cyclohexane fractions were evaporated under N_2_ at 40 °C, and the residue was dissolved in 250 µl of methanol. After filtration (0.2 µm, Phenex™ PTFE 4 mm syringe filters), samples were loaded into HPLC vials. The samples were applied to an ARC HPLC (Waters) via a reversed-phase column (Innoval C18 (2) 150 × 4.6 mm) with 100% methanol (rate flow 1 ml min^−1^), and ergosterol was detected at 282 nm with a UV detector.

### Lipid peroxidation of the fungal and oomycete cultures

Lipid peroxidation was measured with the thiobarbituric acid (TBA) method (Hodges et al. 1999); 250 mg of the fungal cells was homogenized with liquid N and with 1 ml 0.1% trichloroacetic acid (TCA), centrifuged and mixed with 20% TCA containing 0.5% TBA, and heated at 95 °C for 30 min. The absorbances were read with a microplate reader (BMGLabtech, ClarioStar) at 532 nm and 600 nm to subtract nonspecific absorption and at 440 nm to subtract sucrose. The results are presented as malondialdehyde (MDA) equivalents (nmol g^−1^ FW).

### Statistics

Differences between the effects of different treatments were compared by analysis of variance (ANOVA), followed by Tukey’s test using a level of significance of *P* < 0.05. When needed, the results were transformed to fulfil the assumptions in the analysis of variance. Correlations were estimated using the Pearson test. All statistical analyses were performed using SPSS software (IBM Statistics, version 29.0.0.0.). In the results and discussion section, we always refer to the differences between the control with solvent (ethanol) and treatments with terpenes.

## Results

### Growth area and ergosterol content

**Fig. 1:**
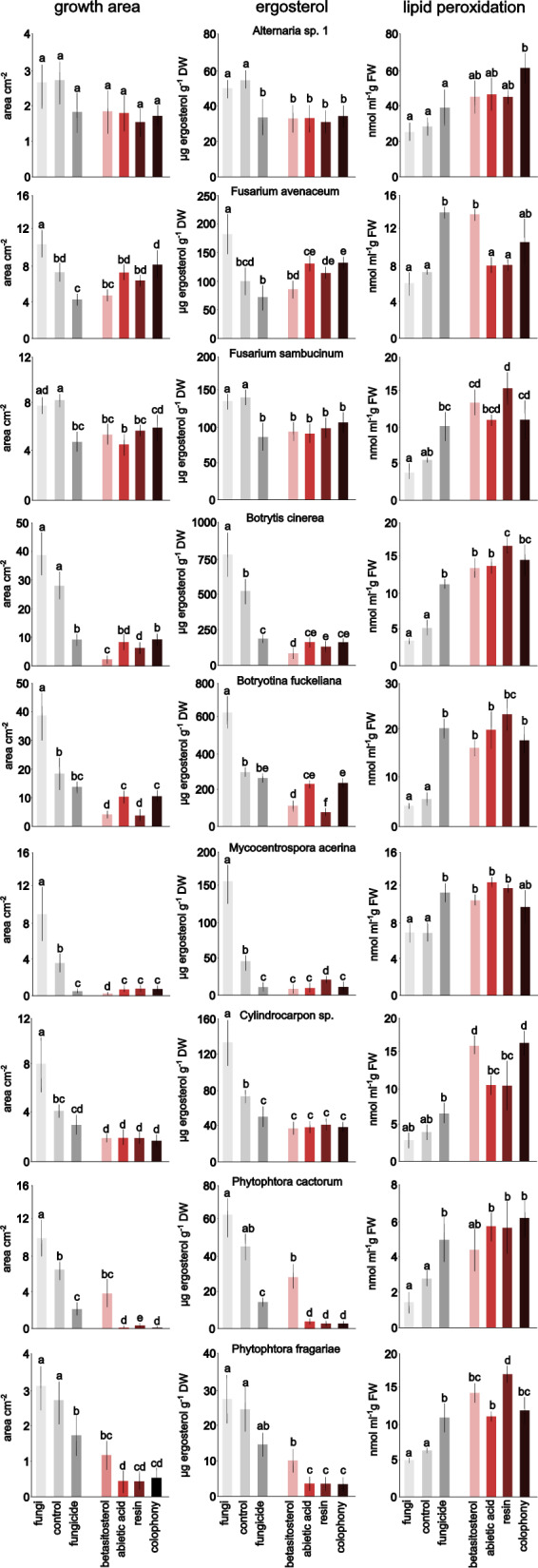
Effect of fungicide and di- and triterpenes on fungi and oomycetes. Left column—growth area, photographed and measured with ImageJ software, middle column – fungal biomass measured with the ergosterol method, right column—lipid peroxidation measured via the malondialdehyde method. The results are presented as the means, and error bars represent standard deviations; results significantly different from each other (p < 0.05) are marked with different letters

Growth area and ergosterol content were affected by treatments in a species- and compound-dependent manner except for *Alternaria* sp., which showed only a slight decrease in area but significant differences in ergosterol concentration for treatments with terpenes (Fig. 1, see Supporting Information for images of fungi and oomycetes). For *F. sambucinum* and *Cylindrocarpon* sp., all terpenes decreased the growth of fungi similarly to fungicide. On the other hand, *F. avenaceum* decreased growth only after the fungicide was added. For the other studied species, the effect caused by the treatments was stronger. For some terpene treatments, the decrease in growth was even more pronounced than that for fungicides (beta-sitosterol for *B. cinerea*, *M. acerina*, and *B. fuckeliana;* abietic acid, colophony and resin for oomycetes).

The minimum inhibitory concentration for beta-sitosterol was 500 ppm, and for other terpenes, it was 200 ppm. The minimum fungicidal concentration was 500 ppm for all terpenes.

### Lipid peroxidation

Peroxidation of lipids of fungal and oomycete cells was dependent on the treatment and studied species. Similar to growth area and ergosterol concentration, lipid peroxidation in *Alternaria* sp. cells was least affected by terpene treatments, showing significantly higher results than that of the control only for colophony. Additionally, for *F. avenaceum,* lipid peroxidation was above the level of the control for only one terpene treatment, beta-sitosterol. However, for the rest of the fungal and oomycete species, the peroxidation of lipids was always higher than that in the control. The highest results were observed with resin for *B. cinerea* and *B. fuckeliana*, with beta-sitosterol for *Cylindrocarpon* sp. and colophony and with resin for *P. fragariae*. Fungicide also increased peroxidation of lipids, but its effect was weaker than that of resin (for *B. cinerea* and *B. fuckeliana*, *P. fragariae*) or beta-sitosterol and colophony (for *Cylindrocarpon* sp.).

### Correlations

For all species the area of growth was positively correlated with ergosterol concentration (Table 2). For most fungi and oomycetes, ergosterol (and growth area) was significantly but negatively correlated with lipid peroxidation (p < 0.001). However, this negative correlation between area and lipid peroxidation was nonsignificant for *Alternaria* sp. (p < 0.1).

**Table 2:** Correlations between growth area, ergosterol and lipid peroxidation. Values represent the correlation coefficient (r) and significance (p) in brackets

Species	Growth area and ergosterol	Ergosterol and lipid peroxidation	Growth area and lipid peroxidation
*Alternaria* sp.	0.921 (0.001)	− 0.504 (0.02)	− 0.366 (0.103)
*F. avenaceum*	0.968 (0.001)	− 0.670 (0.001)	− 0.701 (0.001)
*F. sambucinum*	0.882 (0.001)	− 0.496 (0.022)	− 0.594 (0.005)
*B. cinerea*	0.940 (0.001)	− 0.778 (0.001)	− 0.693 (0.001)
*B. fuckeliana*	0.989(0.001)	− 0.774 (0.001)	− 0.775 (0.001)
*M. acerina*	0.991 (0.001)	− 0.661 (0.001)	− 0.697 (0.001)
*Cylindrocarpon* sp.	0.983 (0.001)	− 0.712 (0.001)	− 0.692 (0.001)
*P. cactorum*	0.997 (0.001)	− 0.816 (0.001)	− 0.809 (0.001)
*P. fragariae*	0.998 (0.001)	− 0.766 (0.001)	− 0.759 (0.001)

## Discussion

Di- and triterpenes showed inhibitory potential against fungi and oomycetes. However, terpenes and fungicide did not decrease the growth of *Alternaria* sp., and the negative effect on *Fusarium spp.* growth, although significant, was weaker than that of other species. It is known that the structure of the fungal cell wall is among the elements that determine microbial resistance (Lima et al. 2019). The cell-wall constituent melanin is a virulence factor for pathogenic fungi (Cousin et al. 2006; Cordero and Casadevall 2017). Melanin distribution and quantity vary widely between species (Nosanchuk et al. 2015). It was shown that loss of melanization increases the fungicide sensitivity of *A. alternata* (Kawamura et al. 1997; Yago et al. 2011). Thus, compared to other species, *Alternaria* spp. and *Fusarium* spp. with melanized mycelia and well-developed cell walls (Yago et al. 2011) were less susceptible to terpenes, as demonstrated in our study. Furthermore, the high susceptibility of oomycetes to terpenes may result from an atypical cell wall structure. Compared to “true” fungi, chitin is only a minor component of oomycete cell walls (Latijnhouwers et al. 2003). Thus, these crucial differences in cell wall structure may explain the stronger effect of terpenes (*Phytophthora* spp.) or weaker effects (*Alternaria* sp., *Fusarium* spp.).

As proposed by Kumari et al. (2019), phenolics and aldehydic terpenes block fungal growth by inhibiting ergosterol biosynthesis. Ergosterol (5,7-diene oxysterol) regulates the permeability and fluidity of cell membranes (Douglas and Konopka 2014), and thus ergosterol is crucial for fungal cell longevity. In our study, decreased growth of fungi correlated positively with a decrease in ergosterol concentration, suggesting that ergosterol biosynthesis is inhibited by di- and triterpenes. For some fungal species, fungal growth was decreased more by beta-sitosterol than other terpenes. The high antifungal efficacy of beta-sitosterol may lie in its chemical analogy to ergosterol; thus, beta-sitosterol may compete with ergosterol for embedding into cell membranes. Similar to beta-sitosterol, resin showed high efficiency in decreasing fungal growth. As resin contains sterols (Bäck et al. 2010), resin may act correspondingly to beta-sitosterol, affecting the ability of ergosterol to embed in cell membranes. On the other hand, the main mechanism behind the inhibition of fungal growth by diterpenes may be different from the mechanism of beta-sitosterol (triterpene) and resin. Diterpenes (abietic acid and a mixture of diterpenes and colophony) may act mainly by inhibiting enzyme activities. It was shown that abietic acid and colophony inhibit some enzymes, i.e., protease, acid sulfatase and acid phosphatase, more than beta-sitosterol (Adamczyk et al. 2015). Moreover, Kumar et al. (2019) showed that phenolics and aldehydic terpenes increase the level of reactive oxygen species (ROS) in cells, leading to increased lipid peroxidation and activation of antioxidant defence systems. In our study, we also observed elevated levels of lipid peroxidation, which suggests that di- and triterpenes utilize analogous ROS-dependent routes of action.

The mechanism underlying the activity of synthetic fungicide fosetyl-Al involves the inhibition of enzymes for which phosphate acts as an allosteric regulator (Fenn 1984), e.g., pyruvate kinases (Johnsen et al. 2019). Moreover, fosetyl-Al induces plant defence mechanisms against pathogens; however, this is beyond the scope of our screening study, as we did not use plants. In contrast to fosetyl-Al, terpenes may act via inhibition or ergosterol, the most abundant sterol in fungal cell membranes, which regulates permeability and fluidity. Moreover, ergosterol is commonly produced by fungi (Kandeler 2015). Thus, inhibition of ergosterol synthesis by terpenes could be a very selective antifungal strategy in contrast to the mode of action utilized by fosetyl-Al. Moreover, fosetyl-Al may cause negative changes in plant yield or propagation, as observed for other synthetic fungicides. For example, fosetyl-Al changes the morphological structures of tomato (*Lycopersicon esculentum* Mill.) pollens (Çali 2008). On the other hand, terpenes, as natural plant products abundant in wood, should not cause adverse effects on plant growth, yield and propagation. Indeed, wood-derived organic amendments favour saprotrophic over pathogenic fungi (Clocchiatti et al. 2021), and forest litter amendments decreased *Fusarium* infections of wheat (Ridout and Newcombe 2016). Coniferous bark contains a particularly large number of terpenes and phenolic compounds (Smolander et al. 2012) and was recently shown to diversify microbiota in agricultural soil (Peltoniemi et al. 2023). Although high production of terpenes is typical for forest trees, agricultural plants, such as strawberry or carrot, also produce terpenes that contribute to their typical flavour and aroma and influence bitterness (Pichersky and Gershenzon 2002). Thus, terpenes may play a role as selective antifungal compounds with no adverse effects on plant growth.

The next step in the development of terpenes as potential biocides is to perform studies with plants. At this stage, measurements should include not only antifungal activity but also the potential effect of terpenes on plant health with the use of rapidly developing tools of metabolomics (Figueiredo et al. 2022). Furthermore, there is a need to develop methods of terpene application in field conditions. Application could include encapsulating terpenes within nanoparticles (Charoenputtakun et al. 2014). This technology is used in biomedical applications to protect active compounds against degradation, allowing them to reach their target in the most effective manner (Di Santo et al. 2021; Venkata et al. 2022). Field studies should include the effect of terpene application on plant and soil health. Finally, follow-up studies should elucidate the potential development of fungal resistance towards terpene-based biocides.

## Conclusions

In conclusion, terpenes showed the potential to act as effective biocides. Our results suggest that terpenes function through inhibiting ergosterol biosynthesis or by preventing ergosterol from embedding into cell membranes. Moreover, terpenes also affect the redox stage in fungal cells, resulting in an increase in lipid peroxidation. The strong negative correlation between growth and lipid peroxidation suggests that the deterioration of lipids may act as a second route for the inhibitory effect of terpenes on fungi. In our laboratory screening study, the effectiveness of terpenes as biocides was at least at the level of the synthetic fungicide fosetyl-Al. Further studies should provide in-depth explanations of the mechanisms behind the action of terpenes on fungi to select the most promising terpenes for studies with plants.

### Supplementary Information

Below is the link to the electronic supplementary material.

Supplementary file1 (DOCX 539 KB)

## Data Availability

The datasets generated during and/or analysed during the current study are available from the corresponding author on request.

## Declarations

### Supporting information

Supporting information Images of fungi and oomycetes growing in Petri dishes.
