# Supplementary figures and images for: Diterpenes and triterpenes show potential as biocides against pathogenic fungi and oomycetes: a screening study
Source: Biotechnol Lett. 2023 Nov 1;45(11-12):1555–63. doi: 10.1007/s10529-023-03438-z (PMC10635980; doi:10.1007/s10529-023-03438-z)

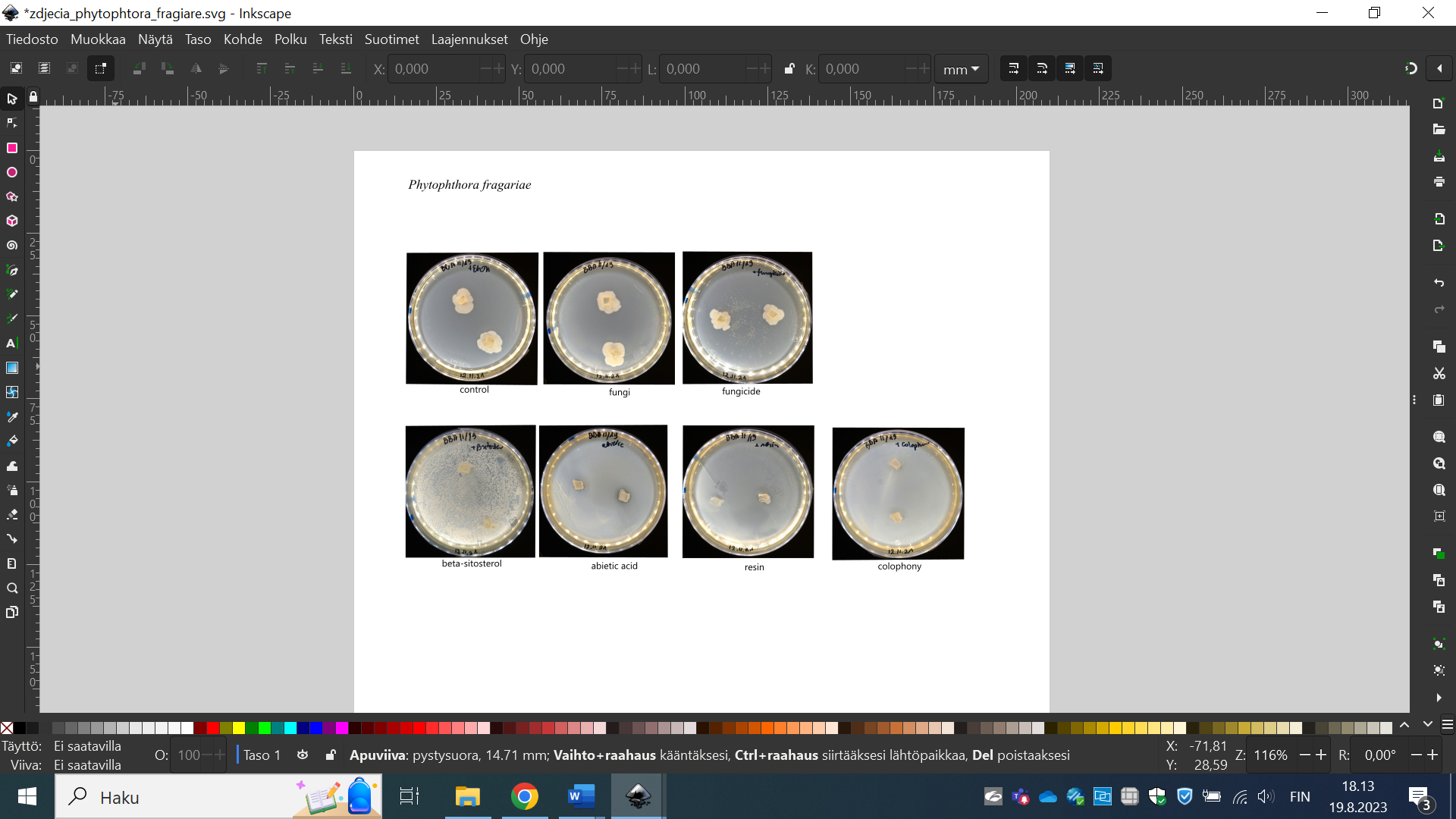

Supplement: Supplementary file 1 — Supplementary file1 (DOCX 539 KB) [file 10529_2023_3438_MOESM1_ESM.docx]
